# TiO_2_—MoS_2_—PMMA Nanocomposites for an Efficient Water Remediation

**DOI:** 10.3390/polym16091200

**Published:** 2024-04-25

**Authors:** Vanessa Spanò, Maria Cantarella, Massimo Zimbone, Federico Giuffrida, Gianfranco Sfuncia, Giuseppe Nicotra, Alessandra Alberti, Silvia Scalese, Libera Vitiello, Sabrina Carola Carroccio, Giuliana Impellizzeri

**Affiliations:** 1 Consiglio Nazionale delle Ricerche, Istituto per la Microelettronica e Microsistemi, CNR-IMM, Via S. Sofia 64, 95123 Catania, Italy; vanessaspano23@gmail.com (V.S.); massimo.zimbone@ct.infn.it (M.Z.); federico.giuffrida@dfa.unict.it (F.G.); giuliana.impellizzeri@ct.infn.it (G.I.); 2 Dipartimento di Fisica e Astronomia, Università di Catania, Via Santa Sofia 64, 95123 Catania, Italy; 3 Consiglio Nazionale delle Ricerche, Istituto per la Microelettronica e Microsistemi, CNR-IMM, Zona Industriale Strada VIII n. 5, 95121 Catania, Italy; gianfranco.sfuncia@cnr.it (G.S.); giuseppe.nicotra@cnr.it (G.N.); alessandra.alberti@cnr.it (A.A.); silvia.scalese@cnr.it (S.S.); 4 Consiglio Nazionale delle Ricerche, Istituto per i Polimeri Compositi e Biomateriali, CNR-IPCB, Via Paolo Gaifami 18, 95126 Catania, Italy; libera.vitiello@cnr.it (L.V.); sabrinacarola.carroccio@cnr.it (S.C.C.)

**Keywords:** titanium dioxide, molybdenum disulfide, nanomaterials, polymer, photocatalysis, water treatment

## Abstract

An improvement of water supply and sanitation and better management of water resources, especially in terms of water reuse, is one of the priorities of the European Green Deal. In this context, it is crucial to find new strategies to recycle wastewater efficiently in a low-cost and eco-friendly manner. The immobilization of inorganic nanomaterials on polymeric matrices has been drawing a lot of attention in recent years due to the extraordinary properties characterizing the as-obtained nanocomposites. The hybrid materials, indeed, combine the properties of the polymers, such as flexibility, low cost, mechanical stability, high durability, and ease of availability, with the properties of the inorganic counterpart. In particular, if the inorganic fillers are nanostructured photocatalysts, the materials will be able to utilize the energy delivered by light to catalyze chemical reactions for efficient wastewater treatment. Additionally, with the anchoring of the nanomaterials to the polymers, the dispersion of the nanomaterials in the environment is prevented, thus overcoming one of the main limits that impede the application of nanostructured photocatalysts on a large scale. In this work, we will present nanocomposites made of polymers, i.e., polymethyl methacrylate (PMMA), and photocatalytic semiconductors, i.e., TiO_2_ nanoparticles (Evonik). MoS_2_ nanoflakes were also added as co-catalysts to improve the photocatalytic performance of the TiO_2_. The hybrid materials were prepared using the sonication and solution casting method. The nanocomposites were deeply characterized, and their remarkable photocatalytic abilities were evaluated by the degradation of two common water pollutants: methyl orange and diclofenac. The relevance of the obtained results will be discussed, opening the route for the application of these materials in photocatalysis and especially for novel wastewater remediation.

## 1. Introduction

Among the environmental problems, water pollution is an urgent topic that needs to be addressed [1]. If, on the one hand, industrial development has brought improvements in the quality of human life, on the other hand, it has caused environmental devastation, including water resources. In addition, the increasing global population implies a growing demand for a greater quantity and quality of water compared to the past. One potential solution to the clean water supply problem is the reuse of wastewater [2]. However, this approach becomes challenging due to the presence of dangerous substances, suspended particles, or organic compounds that are difficult to treat and remove using conventional water treatment methodologies. In addition, some other effective methods have several drawbacks; for example, chlorination generates toxic by-products, while ozonation, although it was a very rapid method and also able to destroy viruses and bacteria, is not economical and very corrosive [3]. Consequently, finding new methods to remove pollutants from water resources has become essential [4].

In the field of wastewater treatment technologies, advanced oxidation processes (AOPs) have garnered increasing attention in recent years. The success of AOPs relies on the production of reactive free radicals (such as hydroxyl, superoxide, and hydroperoxyl radicals) active in the degradation of persistent organic pollutants (POPs), microorganisms (like bacteria), and disinfection residuals [5,6]. This phenomenon occurs because the radicals involved in AOPs have a high oxidation potential that allows for the efficient degradation of organic substances and the killing of bacteria.

One of the most common AOPs used for the photodegradation of organic water pollutants is heterogeneous photocatalysis [7,8,9]. It is a process in which a solid semiconductor is exposed to electromagnetic radiation with energy equal to or greater than the material’s band-gap energy; the irradiation promotes an electron from the valence band to the conduction band, leaving a hole in the valence band. The generated free carriers can migrate to the photocatalyst’s surface, where they can interact with the molecules adsorbed on it. While the holes can oxidize the water molecules and produce hydroxyl radicals (^●^OH), the electrons have the power to reduce the electron acceptors, like oxygen molecules, to form superoxide radicals (^●^O_2_^−^). These reactive species are able to degrade the organic contaminants and reduce bacterial growth in wastewater [7,8,9].

In this field, titanium dioxide (TiO_2_), also known as *titania*, has emerged as a valid photocatalytic material due to its biological and chemical inertness, robust oxidation ability, and long-term durability against photo and chemical corrosion [10,11].

The recombination of photo-generated charges constitutes one of the main drawbacks of photocatalysis, which must be suppressed to improve the activity of the photocatalyst [12]. Studies on photocatalysis are mostly focused on strategies to improve photocatalytic activity, such as heterojunction formation due to the presence of additional semiconductors [13].

Layered two-dimensional (2D) transition metal dichalcogenides (TMDs), such as molybdenum disulfide (MoS_2_), have received great interest in recent years in the scientific community due to some of their intrinsic properties, such as tunable band-gap by changing the number of the layers, optical features, and resistant interaction with light [14,15]. The quick recombination of photo-generated charges usually prevents charge transfer to the surface, so their photocatalytic activity is relatively small. However, these materials can act as co-catalysts for TiO_2_ photocatalysts, improving the photocatalytic characteristics of the titania by creating a heterojunction [16,17,18].

The photocatalytic activity can also be enhanced by increasing the photocatalyst’s surface-to-volume ratio [19]. Hence, TiO_2_ nanostructures have a great deal of potential as highly efficient photocatalysts [11,20,21,22,23]. The Evonik TiO_2_ P 25 nanoparticles are the most widely utilized photocatalyst at the moment [24]. However, using this form of TiO_2_ requires an additional step for the recovery of the photocatalysts after the water treatment, requiring time and effort. As a result, a potential way to use nanomaterials in real water treatment applications consists of their immobilization on an inert support, such as a polymeric matrix [25,26,27].

In this paper, poly(methyl methacrylate) (PMMA) was used as an inert support to form a polymeric film nanocomposite in the presence of Evonik Aeroxide^®^ TiO_2_ P 25 nanoparticles, like active photocatalyst, and MoS_2_ nanoflakes, like co-catalyst to enhance the titania’s photocatalytic performance. The choice of PMMA as the polymeric matrix lies in its properties, such as its transparency to visible light, mechanical rigidity, and UV stability [28]. It is also a cost-effective polymer, making it suitable for water applications [28]. The polymeric nanocomposites were produced by applying the simple and inexpensive solution casting process. By this technique, we obtained films with TiO_2_ and MoS_2_ nanoparticles trapped in their surface, which solves the problem of powder dispersion in the environment when they are utilized as free powders for wastewater treatment.

To our knowledge, few studies on polymeric composites made of TiO_2_ and MoS_2_ have been reported in the literature, and only one work is about the use of combined TiO_2_, MoS_2_, and PMMA for photocatalytic application [29,30,31]. However, in our case, the studied nanocomposites are obtained using the simple method of solution casting, which is more appropriate for large-scale applications. In addition, in our samples, the nanomaterials are anchored to a polymeric inert support; as a consequence, no additional step of recovery is required after the water treatment, unlike the samples described by Li et al.

## 2. Experimental Section

### 2.1. Preparation Method

The Aeroxide^®^ TiO_2_ P 25 was bought by Evonik (3–99 nm particle size) (Essen, Germany), while the MoS_2_ powders were purchased from Sigma-Aldrich (90 nm particle size) (St. Louis, MI, USA). The commercial MoS_2_ powders were previously characterized by our group [32]. Scanning electron microscopy evidenced the flake morphology of the powders; in particular, large MoS_2_ flakes (about 1 μm in size) together with small flakes (about 100 nm in size) were observed [32]. Transmission electron microscopy showed MoS_2_ nanoflakes with different areas and shapes stacked on top of each other [32]. In addition, a fast Fourier transform taken on a portion of the specimen showed the regular crystalline nature of the MoS_2_ [32].

The polymeric nanocomposites, the object of this work, were prepared utilizing the sonication and solution casting method, in which the polymer, i.e., the PMMA, was dissolved in acetone, and in another vial, the powders of TiO_2_ and MoS_2_ were dispersed and always sonicated in acetone. The amount of TiO_2_ was fixed at 5% in weight with respect to the polymer, while the amount of MoS_2_ was changed from 10% to 30% in weight with respect to the used titania. After sonicating the nanoparticles and once the polymer was dissolved, the two vials were mixed and subjected to additional sonication. Then, the resulting mixtures were cast into Petri dishes that were placed in a fridge to allow the solvent to evaporate slowly at a temperature of 4 °C.

In order to figure out whether or not the samples with various amounts of molybdenum disulfide had improved the photocatalytic effectiveness, a reference sample only consisting of titanium dioxide was also synthesized. Moreover, a simple PMMA film was produced and used as a reference.

**Figure 1:**
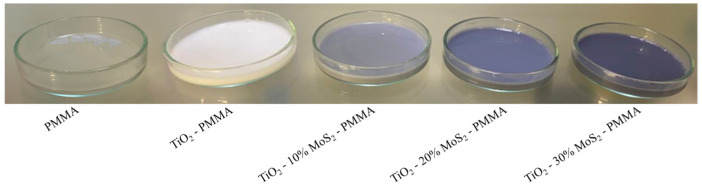
Pictures of the prepared composites: PMMA, TiO_2_—PMMA, TiO_2_—10% MoS_2_—PMMA, TiO_2_—20% MoS_2_—PMMA, TiO_2_—30% MoS_2_—PMMA.

The prepared materials will be hereafter indicated: “PMMA”, “TiO_2_—PMMA”, “TiO_2_—10% MoS_2_—PMMA”, “TiO_2_—20% MoS_2_—PMMA”, “TiO_2_—30% MoS_2_—PMMA”. Figure 1 shows the pictures of the films in the Petri dishes. The PMMA films resulted in transparency, as foreseen for PMMA [28]. The TiO_2_—PMMA appeared white in color due to the presence of the titania, while the presence of MoS_2_ induced in the composites a grey coloration, from light grey to dark grey with increasing the MoS_2_ content (as expected, due to the band-gap energy of the material in the Vis spectrum) [14,15].

### 2.2. Characterizations

Transmission electron microscopy (TEM) was used to characterize the morphology, chemistry, and structure of the nanomaterials. The TEM specimens were prepared by ultramicrotomy. The samples depicted in Figure 1 were embedded in resin and cut by a Leica EM TXP, creating flat slices containing a portion of our nanocomposite. Thin sectioning was performed with a Diatome ultra 35° diamond knife of a Leica EM UC7 ultramicrotome (Wetzlar, Germany), producing 70 nm thin TEM specimens, which were collected on copper TEM grids. To enhance the stability under the electron beam, the TEM specimens were coated with an ultrathin conductive layer of carbon (~3 nm) by a Quorum Q150V ES plus coater(Laughton, East Sussex, UK). A Jeol TEM ARM-200F (Tokyo, Japan) was used at 200 kV for the TEM characterization. The microscope was equipped with a cold field emission gun (FEG) emitter with 0.27 eV energy spread and a Gatan imaging filter QuantumER for electron energy loss spectroscopy (EELS). Scanning mode (S-TEM) was used for imaging and for spectrum imaging (SI) mode, which simultaneously collects spatial and spectroscopic information. Parallel-beam, conventional mode (C-TEM) was used to obtain electron diffraction patterns.

X-ray Crystallographic investigation by X-ray (XRD) was performed by a Rigaku Smartlab diffractometer (Tokyo, Japan) supplied with a rotating anode working at 45 kV-100 mA and of a HyPix 3000 detector (Tokyo, Japan).

XPS analysis was carried out by the PHI Genesis Multi-Technique Scanning XPS system, with a monochromatic Al K_α_ X-ray beam and a 180° hemispherical electron energy analyzer. The system is equipped with a dual-beam charge neutralization system that allows turnkey neutralization of all types of insulating samples.

In order to investigate the optical band-gap of the polymeric nanocomposites, UV-Vis diffuse reflectance spectroscopy (DRS) characterization was conducted by a Perkin-Elmer Lambda 1050+ UV/Vis/NIR spectrophotometer equipped with an integrating sphere. The optical band-gap of the nanocomposites was calculated using the Kubelka-Munk and Tauc-Plot procedure [33].

Thermogravimetric analyses (TGA) were performed using a Perkin Elmer TGA 8000 (Waltham, MA, USA) apparatus to assess the thermal stability of the polymer samples. Samples of 2 ± 0.4 mg were heated under nitrogen flow from 50 °C up to 600 °C with a heating rate of 10 °C/min. The experiment was performed in triplicate.

The wettability of the samples’ surface was characterized using DATAPHYSICS-OCA 15 PRO (Filderstadt, Germany) contact angle measurement equipment. The measurements were performed with drops of water. The average of three measurements in three different regions of each sample were considered.

### 2.3. Photocatalytic Tests

Photocatalysis tests were carried out using methyl orange (MO), a common industrial dye, and diclofenac, a popular nonsteroidal anti-inflammatory drug (NSAID). The tests were performed with 1 cm^2^ squared samples, each immersed in a small cylindrical reactor containing 2 mL of deionized water solution of the contaminant with a concentration of 5.5 × 10^−5^ M. In order to follow the pollutant adsorption and desorption on the surfaces of the photocatalyst and of the beaker, the samples were left in the solution for approximately 60 min under dark conditions. When the equilibrium was reached, the UWAVE LED UV lamp system (emission centered at 365 nm, full width at half maximum of 10 nm, irradiance of 12 mW/cm^2^) was switched on to start the photo-degradation test. At regular time intervals (for a total time of 4 h), the irradiated solutions were measured with a UV-Vis spectrophotometer (Lambda 45 Perkin-Elmer) (Waltham, MA, USA) in a wavelength range of 200–700 nm. The pure pollutant solution without any photocatalyst was also tested as a reference. The degradations of MO and diclofenac were evaluated by the absorbance peak at 464 and 276 nm, respectively, in the Lambert–Beer regime. According to the Langmuir–Hinshelwood model, which assumes that the photocatalysis reaction follows a pseudo-first-order kinetic, the photo-degradation reaction rate (*k*) of the contaminants was calculated by the following equation [7]:

(1) $$ln\frac{C}{C_{0}}=-kt$$

where *t* represents the irradiation time, *C* the concentration of contaminant at the time *t*, and *C*_0_ the initial concentration, that is, the value of the concentration reached after the establishment of adsorption/desorption equilibrium of the pollutant on the surface of the photocatalyst and of the beaker. We also estimated the photonic efficiency, which is useful to compare the process efficiencies of different photocatalytic materials [34]. The photonic efficiency describes the moles of product molecules formed divided by the mole (einstein) of photons at a given wavelength (i.e., 365 nm) in the reactor cell [34]. The photonic efficiency (*ξ*) can be calculated via the following expression [34,35]:

(2) $$\xi =\frac{N_{mol}\;\left(mol\cdot {cm^{-2}\cdot s}^{-1}\right)}{N_{ph\;}(einstein\cdot cm^{-2}\cdot s^{-1})}=\frac{R^{in}}{R_{o},\lambda }\times 100$$

where *R^in^* (mol·cm^−2^·s^−1^) is the initial rate of photo-conversion of the organic molecule, and *R_o_* (Einstein·cm^−2^·s^−1^) is the photon flow [35].

## 3. Results and Discussion

**Figure 2:**
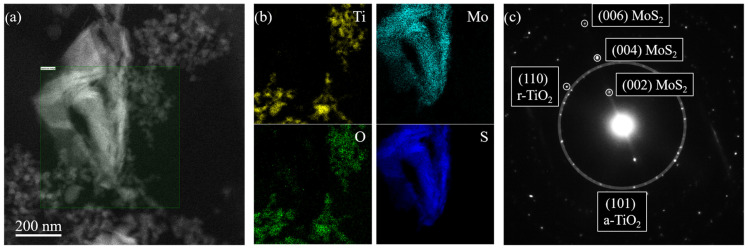
HAADF S-TEM picture of the TiO_2_—10% MoS_2_—PMMA sample (**a**); Ti (yellow), O (green), Mo (cyan), and S (blue) elemental distribution extracted by the fitting of EELS spectra collected in SI mode (**b**); diffraction patterns of the TiO_2_—10% MoS_2_—PMMA sample showing diffraction spots from anatase-TiO_2_ (a-TiO_2_), rutile TiO_2_ (r-TiO_2_), and MoS_2_ (**c**).

Figure 2 reports the TEM characterization of the TiO_2_—10% MoS_2_—PMMA sample. The morphology of the nanomaterials embedded inside the PMMA matrix was studied by S-TEM imaging using a high-angle annular dark-field (HAADF) detector. The result of this analysis is reported in Figure 2a. The HAADF signal intensity is roughly proportional to the square of the atomic number of the species. Hence, MoS_2_ nanoflakes appear brighter than TiO_2_ nanoparticles in Figure 2a. The MoS_2_ nanoflakes have a characteristic size of hundreds of nanometers. The observed structure results from the aggregation and folding of several 2D MoS_2_. The TiO_2_ nanoparticles are much smaller, having a size of the order of a few tens of nanometers. The estimated sizes correspond to the dimensions declared by the manufacturers (the reader can refer to the Section 2). The TiO_2_ nanoparticles decorating the MoS_2_ flakes are close enough to interact with them.

The chemistry of the nanomaterials inside the composite was analyzed by EELS in S-TEM SI mode. An EELS spectrum in the 100–600 eV energy range was collected for every electron probe position inside the green-shaded area depicted in Figure 2a. From the resulting dataset, it was possible to obtain the spatial distributions of Ti, Mo, O, and S (Figure 2b), fitting respectively the Ti L-edge at 456 eV, Mo M-edge at 227 eV, O K-edge at 532 eV, and S L-edge at 165 eV, after background modeling. The obtained elemental distribution makes evident the overlap of Ti/O and Mo/S signals, confirming, respectively, the chemical nature of TiO_2_ nanoparticles and MoS_2_ nanoflakes.

The electron diffraction analysis was used to investigate the crystal structure of the nanomaterials present in the composite. The diffraction patterns reported in Figure 2c showed several spots, both from TiO_2_ and MoS_2_ [36,37]. The larger white-shaded circle encloses the diffraction spots from (101) planes characteristic of the anatase phase of TiO_2_ (a-TiO_2_). A few diffraction spots relative to the (110) planes of the rutile phase of TiO_2_ (r-TiO_2_) were also observed, confirming the mixed-phase nature of the TiO_2_ nanoparticles. The diffraction analysis also showed evenly spaced diffraction spots from (00n) planes of MoS_2_ (with n = 2, 4, 6), characteristic of the stacking direction of the hexagonal MoS_2_ phase.

**Figure 3:**
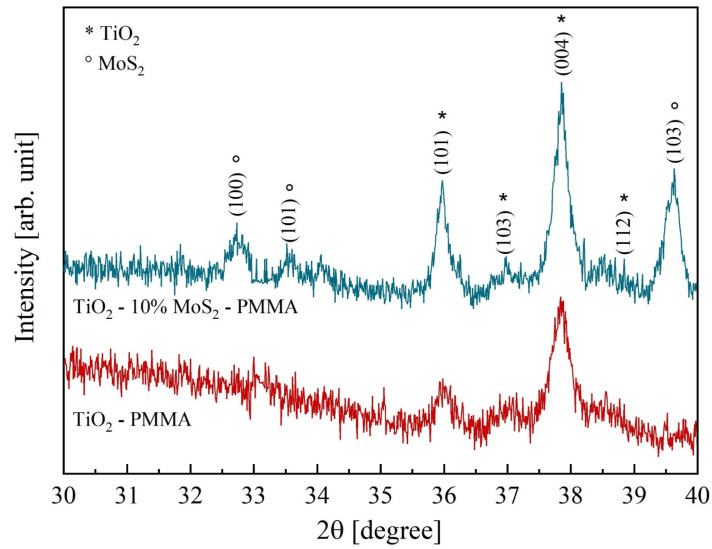
X-ray diffractograms of TiO_2_—PMMA (red curve) and TiO_2_—10% MoS_2_—PMMA (blue curve). The peaks corresponding to TiO_2_ and MoS_2_, respectively, are specified.

Figure 3 reports the diffractograms of the two composites, TiO_2_—PMMA and TiO_2_—10% MoS_2_—PMMA, respectively. In the TiO_2_–PMMA sample (represented with the red curve), most of the TiO_2_ peaks are ascribed to the anatase phase, with minor contributions from rutile. The measured lattice parameters are a = 0.378 nm, b = 0.378 nm, and c = 0.951 nm for TiO_2_ anatase; a = 0.457 nm, b = 0.457 nm, and c = 0.300 nm for TiO_2_ rutile. In the other sample (blue pattern in Figure 3), a blending of the two inorganic materials is visible since, together with the TiO_2_ peaks, the contribution related MoS_2_ is also present with lattice parameters a = 0.315 nm, b = 0.315 nm, and c = 1.232 nm. The TiO_2_ lattice parameters are unchanged with respect to the reference case (i.e., TiO_2_—PMMA sample). The grain size calculated from the full width at half maximum of the main diffraction peaks in TiO_2_ (2θ = 37.84°) and MoS_2_ (2θ = 39.62°) are 38 nm and 34 nm, respectively. In Table 1, the peak position, the interplanar distances, and the associated phases are listed.

**Figure 4:**
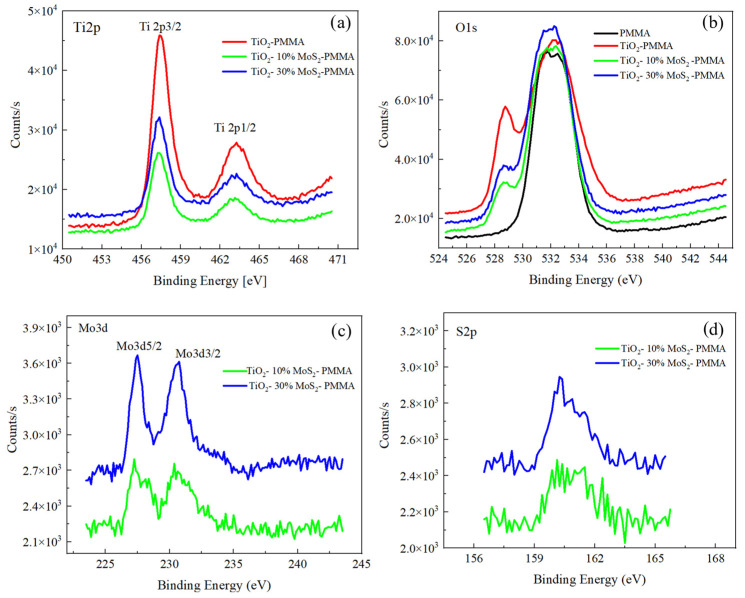
XPS spectra recorded for (**a**) Ti2p, (**b**) O1s, (**c**) Mo3d, and (**d**) S2p for the different samples.

The samples were characterized by XPS to investigate their chemical composition, and the XPS spectra are shown in Figure 4. The Ti2p spectra for TiO_2_—PMMA, TiO_2_—10% MoS_2_—PMMA, and TiO_2_—30% MoS_2_—PMMA are reported in Figure 4a; the O1s spectra for PMMA, TiO_2_—PMMA, TiO_2_—10% MoS_2_—PMMA, and TiO_2_—30% MoS_2_—PMMA are reported in Figure 4b; the Mo3d and S2p spectra for TiO_2_—10% MoS_2_—PMMA, and TiO_2_—30% MoS_2_—PMMA are depicted in Figure 4c and Figure 4d, respectively.

The C1s spectra were also acquired and reported in the Supporting Information (Figure S1). They are mainly related to the PMMA material, with a contribution at 284 eV associated with the C-C bonds and other contributions due to C-O or C=O bonds at 285.5 and 288 eV, respectively. In the case of TiO_2_—PMMA, an additional peak was observed at 289 eV, probably associated with carbonate groups in TiO_2_ [38,39]. In the TiO_2_—PMMA composites containing MoS_2_, the C1s peaks are more similar to the case of the PMMA sample since, in general, the contributions related to TiO_2_ are less evident.

The Ti 2p peaks did not show any significant changes for the three samples with or without the MoS_2_, as expected. The binding energies related to 2p_3/2_ and 2p_1/2_ peaks are 457.4 eV and 463.1 eV for all the samples and are consistent with the standard binding energy found for TiO_2_. The peaks were fitted for TiO_2_—PMMA with four peaks: Ti ^4+^ 2p_1/2_ at 463.1 eV, Ti ^4+^ 2p_3/2_ at 457.4 eV, Ti ^3+^ 2p_1/2_ at 464.1 eV, and Ti ^3+^ 2p_3/2_ at 458.8 eV.

Regarding the O1s peaks, in the case of PMMA, two different contributions have to be taken into account due to the presence of O atoms bound to C atoms within the polymeric structure, with single or double bonds, which correspond, respectively, to binding energies of 531.4 eV and 533 eV. For the PMMA–TiO_2_ sample, the O1s feature also contains other contributions related to the Ti-O bonds, in particular to lattice O, which is found at lower binding energy (528.7 eV), and an additional peak that can be addressed to O vacancy or OH in the TiO_2_ structure.

The Ti2p peaks and the O1s feature at 527 eV, due to the TiO_2_ structure, are more evident in the PMMA–TiO_2_ sample with respect to the samples also containing MoS_2_. Indeed, we expect that the presence of MoS_2_ could hide the TiO_2_ in the composite, determining a lower intensity of the TiO_2_ peaks.

The Mo3d and the S2p spectra reported in Figure 4c,d refer to samples TiO_2_—10% MoS_2_—PMMA and TiO_2_—30% MoS_2_—PMMA, respectively, and show two peaks at 227.5 eV and 230.6 eV for Mo3d_5/2_ and Mo3d_3/2_, respectively, and a large peak for S2p centered at 160 eV, given by the convolution of S2p_3/2_ and S2p_1/2_. The MoS_2_ powder used for the preparation of the nanocomposite materials was also analyzed as a reference, and the spectra are reported in the Supporting Information (Figure S1).

**Figure 5:**
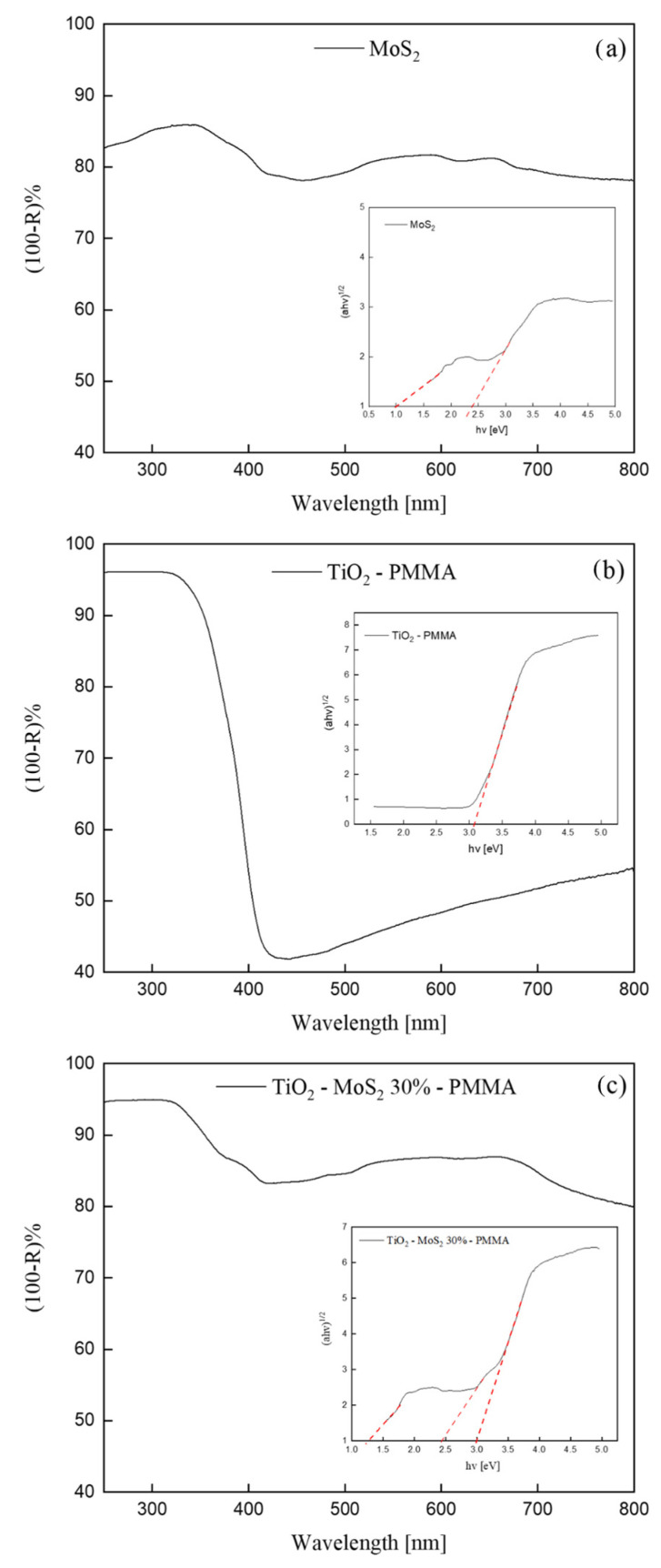
(100-Reflectance)% spectra of MoS_2_ (**a**), TiO_2_—PMMA (**b**), and TiO_2_—30% MoS_2_—PMMA (**c**) sample. The insets of the figures show the Tauc-plots (continuous line) and the relative fit (dashed line) for all the samples.

Figure 5 depicts UV-Vis DRS spectra of pristine MoS_2_, TiO_2_—PMMA, and TiO_2_—30% MoS_2_—PMMA. We reported the apparent absorbance, i.e., (100-Reflectance)%, for convenience.

The MoS_2_ UV-Vis spectrum shown in Figure 5a exhibited absorption peaks in accordance with the literature [40]. The crystal structure of bulk MoS_2_ consists of a vertical arrangement of MoS_2_ layers connected by weak van der Waals forces [15]. The bulk MoS_2_ material is reported to have an indirect band-gap of 1.3 eV [40]. With decreasing the layer thickness, progressive confinement induces a shift of the energy gap from the bulk value of 1.3 eV to over 1.9 eV, together with a change from indirect to direct band-gap in the monolayer limit [40]. Consequently, the various absorption peaks observed in the UV-Vis spectrum (Figure 5a) can be correlated to MoS_2_ nanoflakes with different thicknesses.

The UV-Vis spectrum of the TiO_2_—PMMA sample (Figure 5b) displayed an absorption at wavelengths lower than 400 nm that is consistent with the reported bad-gap energy of the TiO_2_ P 25 (that has a mixed anatase and rutile phase) [41].

In Figure 5c, the UV-Vis spectrum of the TiO_2_—30% MoS_2_—PMMA nanocomposite is reported. The spectrum reasonably shows both the features of TiO_2_ and MoS_2_. Indeed, at ~400 nm, the reader can see the absorption related to the TiO_2_, while the rest of the spectrum (at higher wavelengths) can be correlated to the absorption by the MoS_2_ nanomaterials.

The analyses of these spectra were performed using the Kubelka–Munk and Tauc-plot procedure [33] and are reported in the insets. The inset of Figure 5a shows two band-gaps related to the MoS_2_: (1.3 ± 0.1) eV and (2.4 ± 0.2) eV, in accordance with the reported modulation of the band-gap energy with the layer thickness, as discussed above [40]. The Tauc-plot reported in the inset of Figure 5b revealed the presence of the band-gap of the titania at (3.0 ± 0.3) eV, in perfect agreement with the existing literature [41]. The inset of Figure 5c indicates the presence of multiple optical band-gaps related to MoS_2_ and TiO_2_ contributions. The UV-Vis spectra of TiO_2_—10% MoS_2_—PMMA and TiO_2_—20% MoS_2_—PMMA, together with the Tauc-plots, are reported in the Supplementary Information as Figure S2. No significant differences were observed.

**Figure 6:**
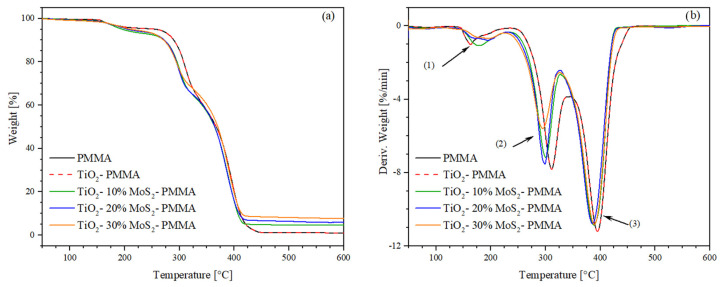
(**a**) Thermogravimetry (TGA) and (**b**) derivative thermogravimetry (DTG) thermograms of the studied samples. The number 1, 2 and 3 represent typical three-step decomposition of PMMA.

The thermogravimetry (TGA) and derivative thermogravimetry (DTG) results are shown in Figure 6a and Figure 6b, respectively. The onset temperature (T_onset_) and peak temperature (T_peak_) of the degradation stages were extrapolated from the DTG curves, while the weight loss and residue at 600 °C were determined from TGA curves. Table 2 shows the extrapolated thermogravimetric data. According to the literature, the PMMA exhibits the typical three-step decomposition. The first mass loss begins at 144 °C and is associated with the cleavage of head-to-head H–H bonds, characterized by a lower bond energy compared to the C–C backbone bond due to steric hindrance and the inductive effect of ester groups. The second mass loss step (T_peak_ = 259 °C) is related to the scission of unsaturated ends triggered by homolytic cleavage of the vinyl group. The final degradation phase partially overlaps with the previous degradation step and is attributed to random chain scissions [42]. The addition of titanium dioxide and molybdenum disulfide did not induce any significant change in the thermal stability of the PMMA during the first-step decomposition. However, in the second degradation phase, MoS_2_ accelerates the thermal degradation of the matrix by approximately 20 °C in the system with the highest filler content (30%).

Considering the potential application of the produced materials in wastewater remediation, the wettability of the surfaces plays an important role. The mean values of the contact angles for each investigated sample are reported in Table 3. The contact angles remained constant within the experimental errors for all the investigated surfaces. The contact angles of about 80° additionally indicated good hydrophilicity of the investigated surfaces [43], which are hence promising for water treatment through photocatalysis.

**Figure 7:**
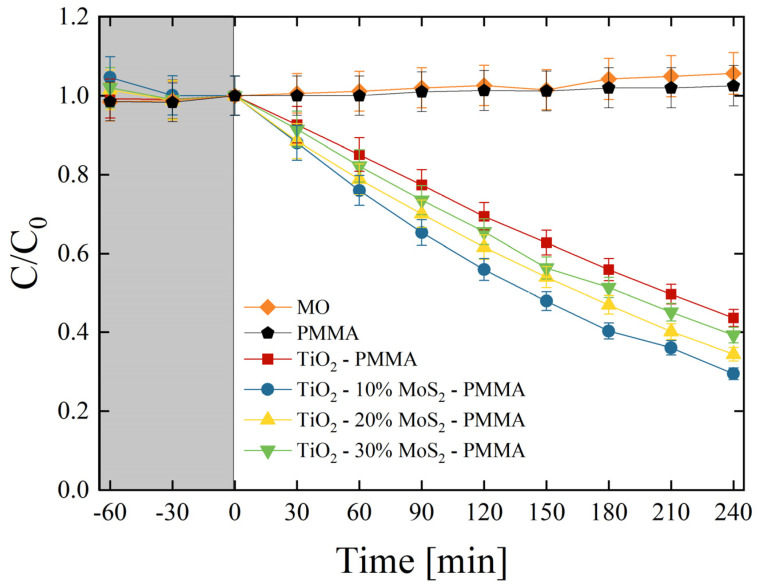
MO photo-degradation under UV light irradiation for six aqueous solutions with MO (diamonds), MO with PMMA (pentagons), MO with TiO_2_—PMMA composite (squares), MO with TiO_2_—10% MoS_2_—PMMA composite (circles), MO with TiO_2_—20% MoS_2_—PMMA composite (up-triangles), MO with TiO_2_—30% MoS_2_—PMMA composite (down-triangles). The grey region indicates the preliminary adsorption test in the dark.

The photocatalytic aptitude of the composites was first tested by the degradation of MO dye. Methyl orange is a synthetic anionic azo dye, frequently used as a colorant in textile and leather industries. It is also widely used in research laboratories as a pH indicator because of its clear color variance at different pH values. MO is a carcinogenic dye, so it must be treated before discharging into the environment [44]. All the produced composites were tested, and the results are reported in Figure 7. *C* is the concentration of MO at the irradiation time *t*, while C_0_ is the starting concentration of MO (as detailed in the Section 2). The preliminary test led in the dark (grey area in Figure 7) allowed an estimation of the adsorption of MO on the surfaces of the samples and on the walls of the cylindrical vessels. The adsorption result was negligible. Under UV light irradiation, the mere MO solution and the MO solution in the presence of only PMMA did not show any variation, as expected. On the other hand, the TiO_2_ P 25 induced a significant degradation of the dye (~55% of the dye is degraded after 240 min of irradiation under UV light). The degradation efficiency is further increased thanks to the presence of MoS_2_ nanoflakes. We observed that the best photocatalyst result was in the TiO_2_—10% MoS_2_—PMMA composite, which is the material with the smallest amount of MoS_2_ (~70% of the dye is degraded after 4 h of UV light irradiation).

In order to quantify the photo-degradation process, the reaction rates (*k*) were estimated by applying the Langmuir–Hinshelwood model, as detailed in the Section 2 [7]. Table 4 reports the photo-degradation reaction rates for all the investigated samples. In detail, the reaction rate increased from (1.51 ± 0.08) × 10^−3^ min^−1^ of the TiO_2_—PMMA composite to (2.20 ± 0.11) × 10^−3^ min^−1^ of the TiO_2_—10% MoS_2_—PMMA composite with a remarkable increase of about 50% thanks to the presence of MoS_2_ co-catalyst.

The photonic efficiency (*ξ*) was calculated (the reader can refer to the Section 2 for the details) for all the investigated samples and is reported in Table 5. The photonic efficiency values in the case of MO (*ξ*_MO_) indicated again the TiO_2_—10% MoS_2_—PMMA as the best performing material, in agreement with the results reported in Figure 5.

The potential leak of the nanomaterials from the polymeric support was investigated by removing the samples from the MO solutions after 240 min under UV lamp. The mere solutions were then irradiated with the UV light for 1 h and no reduction of MO concentration was observed. This experiment demonstrated that not enough nanomaterials were released in the MO solution giving a detectable photo-degradation.

**Figure 8:**
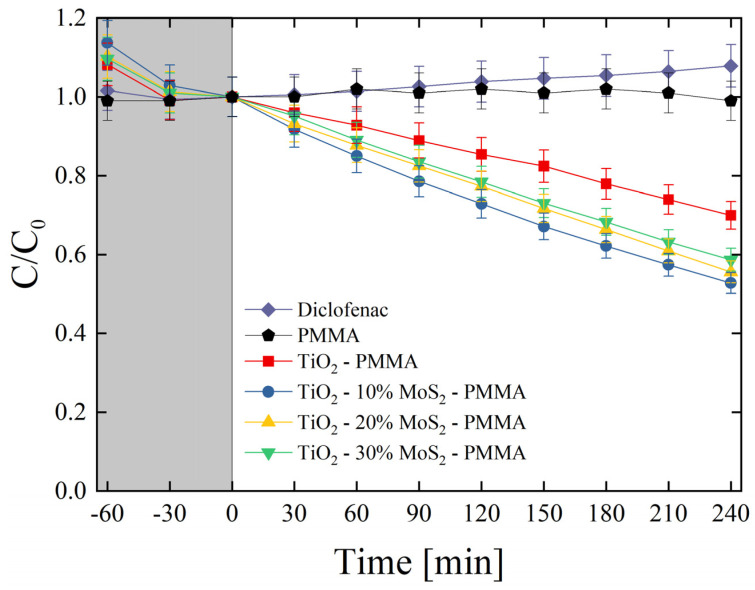
Diclofenac photo-degradation under UV light irradiation for six aqueous solutions with diclofenac (diamonds), diclofenac with PMMA (pentagons), diclofenac with TiO_2_—PMMA composite (squares), diclofenac with TiO_2_—10% MoS_2_—PMMA composite (circles), diclofenac with TiO_2_—20% MoS_2_—PMMA composite (up-triangles), diclofenac with TiO_2_—30% MoS_2_—PMMA composite (down-triangles). The grey region indicates the preliminary adsorption test in the dark.

The produced materials were also tested for the degradation of diclofenac. Diclofenac is an emerging contaminant commonly used as an analgesic for humans, livestock, and domestic animals in the treatment of inflammation and pain in pathologies [45]. The global consumption of diclofenac was estimated at around 940 tons per year, with an average of 65% of this pharmaceutical being released through the urine in the environment [46]. Diclofenac can be toxic for several organisms according to its concentration [47]. It is consequently important to find effective methodologies for the degradation of this drug. Figure 8 reports the photo-degradation of diclofenac by the investigated composites. As expected, no variations in the drug concentration were observed under UV irradiation for the mere diclofenac solution and for the diclofenac solution in the presence of only PMMA. The test evidenced the role of MoS_2_ as a co-catalyst of TiO_2_; indeed, the samples enriched with the MoS_2_ nanoflakes clearly showed an enhanced photocatalytic aptitude with respect to the TiO_2_—PMMA sample. More specifically, the best sample resulted in the TiO_2_—10% MoS_2_—PMMA one (~50% of the drug is degraded after 4 h of UV light irradiation), as in the case of MO degradation (see Figure 7).

The photo-degradation reaction rates (*k*) were estimated for all the studied samples and reported in Table 6. The reaction rate raised from (0.64 ± 0.03) × 10^−3^ min^−1^ of the TiO_2_—PMMA composite to (1.15 ± 0.60) × 10^−3^ min^−1^ of the TiO_2_—10% MoS_2_—PMMA composite with an increase of about 80% thanks to the presence of MoS_2_ co-catalyst.

The photonic efficiency for diclofenac (*ξ*_diclofenac_) was also calculated via Equation (2). Table 7 reports the photon efficiencies of the composites at various wt.% of MoS_2_. Also, for this pollutant, the TiO_2_—10% MoS_2_—PMMA composite showed the highest efficiency compared to all the other samples.

It is worth noting, by comparing the data of Table 4 and Table 6 and Table 5 and Table 7, that the photo-degradation is, in general, lower in the case of diclofenac than MO, surely due to the recalcitrant nature of diclofenac [48].

**Figure 9:**
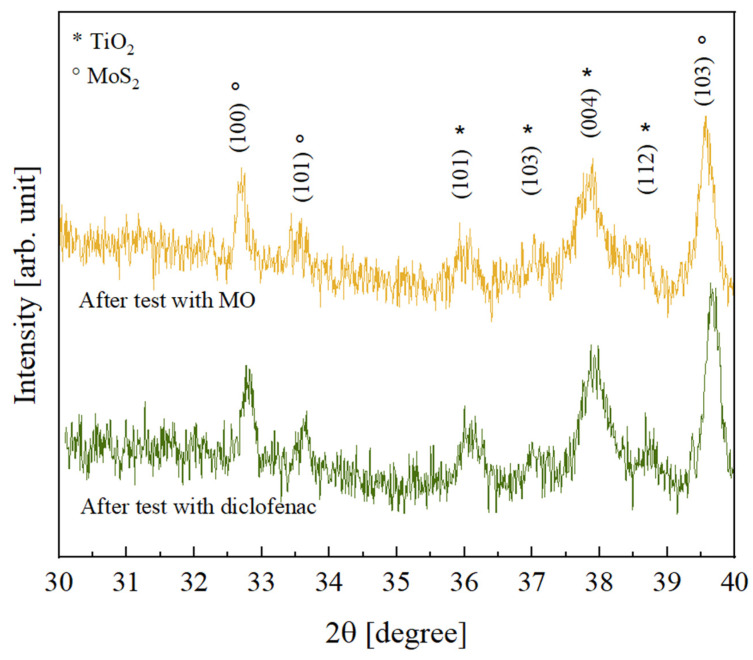
XRD patterns of TiO_2_—10% MoS_2_—PMMA samples after the photocatalytic degradation test of MO (yellow curve) and after the photocatalytic degradation test of diclofenac (green curve).

In order to investigate the possible effects of the photocatalytic process on the crystallinity of the inorganic components, the TiO_2_—10% MoS_2_—PMMA samples after the photocatalytic degradation tests with MO and diclofenac were analyzed by XRD. The results obtained, shown in Figure 9, revealed that no variation was registered; the peaks are indeed comparable to the peaks observed before the photocatalysis (see Figure 3).

The photocatalytic performance of MoS_2_ was also tested under visible light; no activity was revealed surely due to the small band-gap of the material that causes a rapid recombination of the photo-generated electrons and holes.

**Figure 10:**
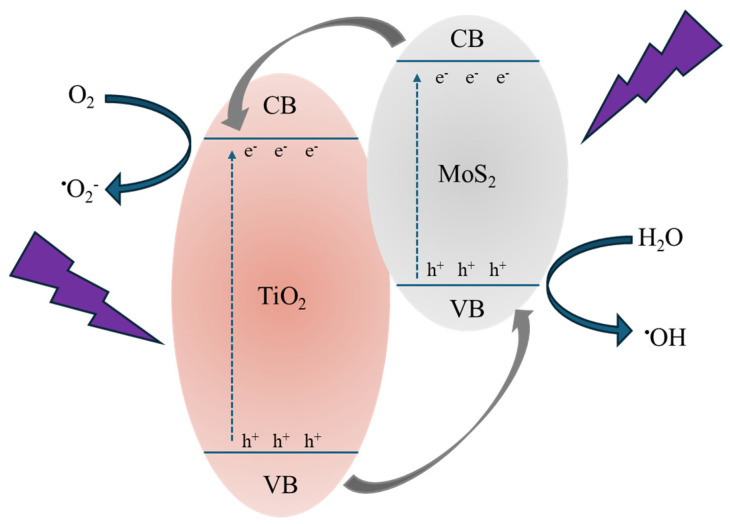
Schematic illustration of the photocatalytic process in the presence of MoS_2_ as co-catalyst.

Combining all the obtained results, we can deduce the crucial effect of MoS_2_ nanoflakes in improving the photocatalytic efficiency of TiO_2_. Indeed, excluding any different role of the various samples’ surfaces (as demonstrated by the contact angle measurements, see Table 3), the observed photocatalytic activity is surely driven by the presence of MoS_2_. Figure 10 reports a tentative description of the acting mechanism. The MoS_2_ can control the electron–hole pair recombination by charge carrier trapping. As reported in the literature [49], the conduction band (CB) and valence band (VB) positions of MoS_2_ are quite higher than those of TiO_2_; as a consequence, under UV irradiation, the photo-excited electrons are transferred from the CB of the MoS_2_ to the CB of the TiO_2_, while the photo-generated holes are transferred from the VB of the TiO_2_ to the VB of the MoS_2_ (see Figure 10). In this way, the recombination of the charge carriers is drastically reduced, obtaining higher photocatalytic performances [50]. Thus, the electrons and holes able to reach the surfaces of the two materials (i.e., TiO_2_ and MoS_2_) initiate a series of redox reactions with oxygen and water molecules adsorbed on the surface, forming reactive radicals (mainly •OH and •O_2_^−^) that react with the organic pollutants, in our tests MO and diclofenac, starting their degradation. The decrease in photocatalytic efficiency with the MoS_2_ powders amount could be understood considering the excessive coverage of the TiO_2_ P 25 surface by the MoS_2_ nanoflakes, which invalidates the photocatalysis process. Indeed, the photocatalytic efficiency of the whole system is driven by a compromise between the MoS_2_ action in separating the charge carriers and the coverage of the TiO_2_ surface resulting from the presence of MoS_2_ nanoflakes that negatively affect the photocatalytic performance of the composites [21,51].

## 4. Conclusions

In this work, we presented TiO_2_—MoS_2_—PMMA nanocomposites obtained by casting from solution. The Evonik Aeroxide^®^ TiO_2_ P 25 photocatalyst was coupled with MoS_2_ nanoflakes as a co-catalyst with the aim of improving the photocatalytic performance of the titania. PMMA was used as a supporting matrix to avoid the release of the nanomaterials in the environment. A detailed TEM characterization demonstrated the intimate contact between the two nanomaterials, TiO_2_ and MoS_2_, which is crucial to improving the photocatalytic aptitude of the titania. The created junction between TiO_2_ and MoS_2_ produces materials with an outstanding photocatalytic performance for the degradation of MO dye and diclofenac drug. The presence of MoS_2_ induces an increment in the photocatalytic activity higher than 50%. In conclusion, the reported results demonstrate that the TiO_2_—MoS_2_—PMMA nanocomposites are promising materials that, overcoming the post-recovery step of nanoparticles after the water treatment, can find applications in wastewater remediation on a large scale.

## Figures and Tables

**Table 1:** Peak position and phase identification taken from the diffraction patterns in Figure 3.

Sample	2θ (°)	Interplanar Distance (nm)	Phase
TiO_2_—PMMA	35.99	0.249	Rutile (101)
	37.08	0.242	Anatase (103)
	37.84	0.238	Anatase (004)
	38.74	0.232	Anatase (112)
TiO_2_—10% MoS_2_—PMMA	32.78	0.273	MoS_2_ (100)
	33.60	0.267	MoS_2_ (101)
	35.96	0.250	Rutile (101)
	37.83	0.238	Anatase (004)
	38.74	0.232	Anatase (112)
	39.62	0.227	MoS_2_ (103)

**Table 2:** Onset temperature (T_onset_), weight loss, peak temperature (T_peak_), and residue at 600 °C of the analyzed samples.

Sample		T_onset_ [°C]	Weight Loss [%]	T_peak_ [°C]	Residue [%]
PMMA and TiO_2_—PMMA	(1)	144	4.6	163	1.2
	(2)	259	34.6	311	
	(3)	340	59.6	395	
TiO_2_—10% MoS_2_—PMMA	(1)	146	6.8	178	4.8
	(2)	255	28.9	300	
	(3)	321	59.5	388	
TiO_2_—20% MoS_2_—PMMA	(1)	145	6.6	195	5.9
	(2)	250	29.6	298	
	(3)	319	57.9	385	
TiO_2_—30% MoS_2_—PMMA	(1)	148	5.5	196	7.9
	(2)	242	26.9	295	
	(3)	323	59.7	389	

**Table 3:** Values of the contact angles for all the studied samples: PMMA, TiO_2_—PMMA, TiO_2_—10% MoS_2_—PMMA, TiO_2_—20% MoS_2_—PMMA, TiO_2_—30% MoS_2_—PMMA.

Sample	Contact Angle (°)
PMMA	82 ± 7
TiO_2_—PMMA	78 ± 8
TiO_2_—10% MoS_2_—PMMA	77 ± 8
TiO_2_—20% MoS_2_—PMMA	76 ± 7
TiO_2_—30% MoS_2_—PMMA	87 ± 9

**Table 4:** Degradation rates of MO at different percentages (0–30%) of MoS_2_ in the samples.

Sample	k_MO_ (min^−1^)
TiO_2_—PMMA	(1.51 ± 0.08) × 10^−3^
TiO_2_—10% MoS_2_—PMMA	(2.20 ± 0.11) × 10^−3^
TiO_2_—20% MoS_2_—PMMA	(1.91 ± 0.10) × 10^−3^
TiO_2_—30% MoS_2_—PMMA	(1.71 ± 0.09) × 10^−3^

**Table 5:** Photonic efficiency for the MO degradation at different percentages (0–30%) of MoS_2_ in the samples.

Sample	*ξ*_MO_ (%)
TiO_2_—PMMA	0.0144 ± 0.001
TiO_2_—10% MoS_2_—PMMA	0.0172 ± 0.001
TiO_2_—20% MoS_2_—PMMA	0.0163 ± 0.001
TiO_2_—30% MoS_2_—PMMA	0.0151 ± 0.001

**Table 6:** Degradation rates of diclofenac at different percentages (0–30%) of MoS_2_ in the samples.

Sample	k_diclofenac_ (min^−1^)
TiO_2_—PMMA	(0.64 ± 0.03) × 10^−3^
TiO_2_—10% MoS_2_—PMMA	(1.15 ± 0.60) × 10^−3^
TiO_2_—20% MoS_2_—PMMA	(1.04 ± 0.06) × 10^−3^
TiO_2_—30% MoS_2_—PMMA	(0.97 ± 0.05) × 10^−3^

**Table 7:** Photonic efficiency for the diclofenac degradation at different percentages (0–30%) of MoS_2_ in the samples.

Sample	*ξ*_diclofenac_ (%)
TiO_2_—PMMA	0.0071 ± 0.001
TiO_2_—10% MoS_2_—PMMA	0.0098 ± 0.001
TiO_2_—20% MoS_2_—PMMA	0.0093 ± 0.001
TiO_2_—30% MoS_2_—PMMA	0.0088 ± 0.001

## Data Availability

The raw data supporting the conclusions of this article will be made available by the authors on request.

## Supplementary Materials

The following supporting information can be downloaded at: https://www.mdpi.com/article/10.3390/polym16091200/s1, Figure S1: (100-Reflectance)% spectra of TiO_2_—10% MoS_2_—PMMA (a), and TiO_2_—20% MoS_2_—PMMA (b) sample. The insets of the figures show the Tauc-plots (continuous line) and the relative fit (dashed line) for all the samples; Figure S2: XPS spectra recorded for (a) C1s for all studied samples; (b) Mo3d for the commercial MoS_2_; (c) S2p for the commercial MoS_2_.
