# Supplementary material for: TiO2—MoS2—PMMA Nanocomposites for an Efficient Water Remediation
Source: Polymers (Basel). 2024 Apr 25;16(9):1200. doi: 10.3390/polym16091200 (PMC11085880; doi:10.3390/polym16091200)
Supplement: Supplementary file 1 [file polymers-16-01200-s001.zip › polymers-2916037-supplementary.pdf]

# Supplementary Information

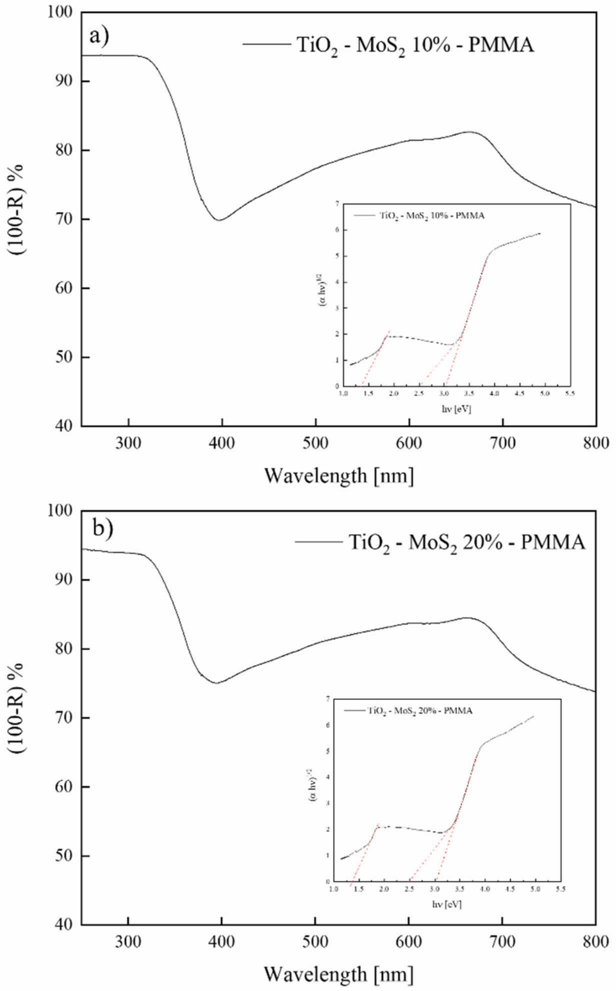


**Figure S1.** (100-Reflectance)% spectra of $TiO_2$ - 10% $MoS_2$ - PMMA (a), and $TiO_2$ - 20% $MoS_2$ - PMMA (b) sample. The insets of the figures show the Tauc-plots (continuous line) and the relative fit (dashed line) for all the samples.

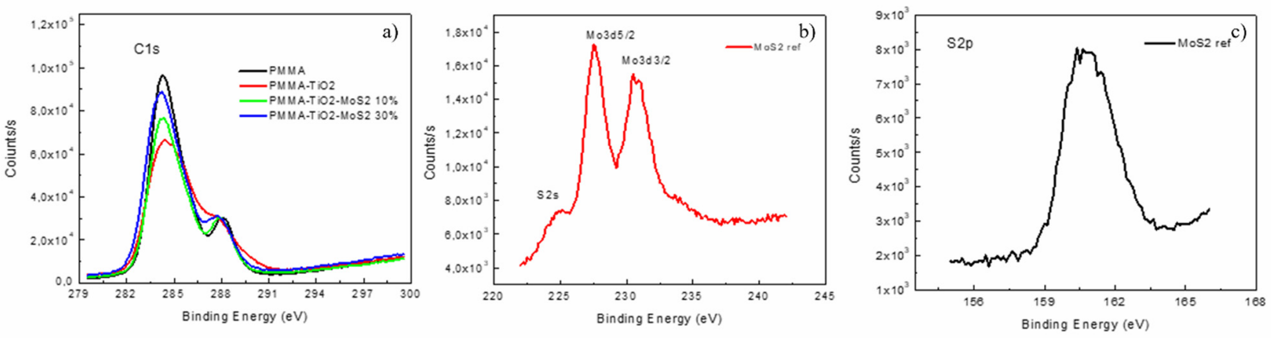


**Figure S2:** XPS spectra recorded for (a) C1s for all studied samples; (b) Mo3d for the commercial $MoS_2$; (c) S2p for the commercial $MoS_2$.
